# Cultivating Systems Leadership: Translating Real‐World Practice Into Classroom Opportunities

**DOI:** 10.1002/yd.70031

**Published:** 2025-11-30

**Authors:** Lucas Díaz, Dwan Adams

**Affiliations:** ^1^ Center for Inclusive Public Life and Tulane University New Orleans USA; ^2^ Tulane University New Orleans USA

## Abstract

This article highlights the benefits of integrating systems leadership approaches into leadership education and pedagogy. Drawing on over 40 years of combined real‐world practice, the authors present “field notes” as “case‐like descriptions and analysis” from their experiences as full participants in community organizing in post‐Katrina New Orleans and global sustainable development with the Peace Corps in Mongolia and Haiti. The field notes demonstrate how an implicit systems leadership mindset was instrumental in achieving outcomes in complex public and international contexts. The article proposes that integrating systems leadership through experiential learning enhances students' abilities to drive sustainable change within complex systems by better preparing students for the intricate, interdependent systems they will encounter beyond the classroom.

## 1 Introduction

Consider this scenario: you are a civically engaged person in your neighborhood who has just walked into the first session of a free 2‐day community organizing training. You don't know what that is, but you trust Jim, the fellow who recruited you. Nametag in hand, you introduce yourself to strangers who were similarly recruited. Then the instructor asks if you read the assignment that was sent earlier. He instructs you to sound off by number, and you end up in one of three groups. He gives you no instructions when he says “begin.”

You and your classmates stare at each other. What are you supposed to do? No one seems to know. Some people start to guess. Others suggest what to do. They start to self‐organize. Someone suggests playing out the reading. Someone else suggests turning the tables. A different instructor removes that person from the room. More people suggest bold moves. They are also removed from the room. After three rounds of this incomprehensible exercise, your emotions are wound tight. You want to complain. Some in the room cry.

Years later, however, you remember this classroom exercise as the first day you opened your eyes to the way power systems operate in the world. Today it is the lesson that still resonates deeply in your leadership practice. As unorthodox as the pedagogy above may appear, it is one that instantly and (perhaps too) abruptly brings into the classroom the uncertain yet felt interaction of power in public systems.

Imagine yourself in a classroom about systems leadership. What expectations would you have for how the class should be conducted? Do you wonder if the classroom will bring sufficient uncertainty, similar to that experienced in real‐world activities? Do you anticipate some risk‐taking, such that moves will be weighed readily against existing and anticipated risks? Will there be sufficient exploration of the connectivity to the proximal and distant leverage points that could or may exert power over your desired actions?

The scenario we opened with certainly offered much (if not all) of the elements we imagined you might ask, but it also offered the potential for harm. We're certainly not advocating that such a teaching approach be replicated, not even with seasoned students. We think, however, that opportunities for more real‐world experiences combined with teaching systems leadership approaches could be beneficial to all leadership educators.

Our combined 40‐plus years of real‐world practice with systems approaches in the field inform us that the more tangible the experience can be in the classroom, the deeper the lesson will sink in. Before we even knew the concept of systems leadership as an approach to leadership practice, we were immersed in real‐world systems leadership learning and action. In this article, we bring attention to the benefit that program‐based leadership education could derive from including more systems leadership approaches (Day and Dannhäuser 2024). We draw from community organizing practices in New Orleans and global sustainable development experiences in the Peace Corps to explore the critical role that systems leadership played in our work in real‐world situations.

Although we did not label the work we describe in the field notes below as systems leadership approaches at the time, it was a way of planning and doing action that was already present and instrumental to solution‐finding within our experiences. For us, each field note presents “case‐like” descriptions and analysis that highlights the role that systems leadership approaches, such as adaptability, engagement, mapping, expanded mental models, and resilience played (even when we didn't fully know these formal labels) played in our abilities to pursue change efforts.

Community organizing field notes below illustrate how systems leadership approaches of adaptability, engagement and mapping enabled understanding of and engagement with interlocking governance systems to secure resources and enact policy changes in New Orleans and across Louisiana. Similarly, global sustainable development field notes underscore the critical role that adaptability, engagement, and expanded mental models played in strengthening local capacity. We identify several key concepts promoted by a systems leadership framework: adaptiveness, engagement, mapping, expanded mental models, and resilience. We argue integrating these components through experiential learning, such as simulations, case studies, and community‐engaged opportunities, along with fostering cultural humility and global awareness, can significantly enhance students' abilities to collaborate effectively and drive sustainable change within complex systems. We conclude by advocating for pedagogical strategies that bridge classroom instruction with real‐world practice and reflection to cultivate systems leadership capacities within students.

### 1.1 Statement on Positionality

Both of our field notes below are derived from the standpoint of full participants in the activities and actions we describe. For the field notes of my community organizing experiences (Lucas), my positionality as an immigrant to the US played a significant role in my post‐Katrina New Orleans work. For the field notes of my global development experiences (Dwan), my positionality as a New Orleanian engaged directly in and with communities abroad informs my assessments about bringing global systems perspectives to local contexts.

## 2 Community Organizing Field Notes

Community organizing, when referenced in this article, refers specifically to the type and style of organizing developed by the Industrial Areas Foundation (IAF n.d.). Today, central features of community organizing include broad‐based organizing, local leadership development, direct action and conflict, and pragmatic approaches (as distinct from ideological positioning and conflict). Professional organizers in the IAF tradition bring community leaders and followers together at the local level, help them direct their energy toward an agreed‐upon problem, conduct research on the problem, then plan an action (or set of actions) to address the problem, which they then execute and evaluate. They repeat this cycle for each action they decide to take, necessary for navigating complex, dynamic systems that impact a group's desired outcomes.

The process described above was derived from Saul Alinsky, regarded as the father of modern‐day community organizing, who created an entire ecosystem, bringing into existence professional methodologies underpinned by theoretical foundations about power, public life, and the common good (Alinsky 1946, 1972; Schutz and Sandy 2011). Framing Alinsky's approach is John Dewey's pragmatist theory, which makes experiential knowledge central to building a person's know‐how within and about the systems in which they engage (Dewey 1927/2016, 2017; Díaz 2024; Schutz and Sandy 2011), an approach echoed by Marshall Ganz today (2009).

### 2.1 Initial Exposure

Professional community organizers across the US who work to organize local communities are highly likely to have been trained by any number of professional development “schools” that can be traced back to IAF (Schutz and Sandy 2011). In my case, I (Lucas) was recruited by New Orleans‐based IAF organizers because I had launched a nonprofit organization serving the newcomer Latinx community. Though I couldn't name it at the time, I was exposed immediately to IAF's systems leadership approaches in New Orleans when the local IAF organized on the issue of rental assistance after the storm. Being completely green to the situation, I participated as a newly recruited community leader in one of the local IAF “house” meetings at a local church. I listened quietly as the group planned the final steps of a significant request they had made to the State of Louisiana's Road Home Program. Weeks later, I joined about 50 people at the State Capitol for a Road Home hearing. In this hearing, a decision to approve $50M for renters (Allen 2008) was made in the group's favor, marking the culmination of a major undertaking by non‐housing experts and non‐political operatives.

What I didn't know at the time–but would later put to practice–was that this group of seemingly random people represented in that moment months of strategic, methodical leadership practice that included mapping the power systems and engaging officials and communities in those systems directly. When the IAF organizes in local communities, it does not come in and announce itself as a resource that helps people fight a target issue. Instead, it engages by building relationships, listening to people, exploring possibilities and then prioritizing with them the top issue goal(s) to pursue. This occurs without technical, issue, or policy experts; these are just regular people debating what they perceive as something that could be better. Next, they research, and they do so deeply. They engage different parties to educate them and engage different audiences on the matter, exploring as many system touch points to the issue as they can perceive and/or imagine. These strategies are used by communities working with IAF organizers, which is like what Drier et al. (2019) described as “look and learn”—a key collaborative stage of systems change (p. 21).

IAF organized community groups incorporate systems leadership approaches of engagement and mapping in their research, analysis, and strategy building. They conduct a power analysis of as many intersecting power levers as they can identify that interact with their target goal (see Tang in references for a representative tool). This means they map out all the known decision‐makers, or levels of authority, at all levels, who can approve or derail their eventual proposal. This is followed by engaging directly with decision‐makers and other influential parties who are meaningfully related to their target goal. From this mapping and engagement work, they make decisions informed by the systems they have learned about and how these interrelate. For the group that secured the Road Home decision, this was the work they conducted prior to the hearing in which I participated. They built relationships with power players in different systems in the region at various municipal, parish (equivalent of county), state, and federal levels in Southeast Louisiana.

In retrospect, it may seem obvious that a local community‐based group would pursue fund allocations for a demographic in‐need from a state agency propped up to distribute resources for rebuilding after the storm. It would be more astute to recognize that a decision to set aside $50M for such a targeted program does not happen easily when it wasn't included in the initial public rollout of the program. It certainly doesn't happen without many different voices across various interlocking and interrelated systems chiming in (both in favor and in opposition).

It is doubtful that without a systems approach, this community‐based group would have succeeded in securing resources for its target demographic in New Orleans. No special allocation request would have been approved by the Road Home program if it didn't have buy‐in from the competing parishes (counties) vying for those dollars, the various agencies with oversight of the program, the political stakeholders across the state with interest in the program, not to mention the interests and vigilance of federal agencies and the general public. For the IAF‐trained community leaders who conducted this work, one can point to the power analysis of systems that enabled them to see the interconnected relationships affecting their desired goals. This mapping work enabled them to put to practice engagement strategies with allies in key areas and helped them create a people‐centered solution that produced general public support.

Although I never heard the term “systems leadership” in my community organizing trainings and experiences, it turns out that a systems approach is necessary in public action for public good. Such an approach of working across systems and engaging players in different systems helps leaders secure support and/or agreement of various power brokers across disparate networks as they work toward acting on a solution for the common good (Warren 2001). For example, without a systems view, power analysis becomes ineffective.

### 2.2 Coordinating Across Systems

As a non‐profit leader, I engaged in various coalitions. I coordinated one such coalition, between 2009 and 2011, which was focused on language access issues for limited English‐proficient people. It was a small group that worked on ensuring due process for limited English‐speaking defendants in New Orleans criminal courts during the Katrina recovery years.

This group represented a variety of civil society allies that were concerned with the civil rights of newcomer migrant laborers in post‐Katrina recovery New Orleans. They perceived that the civil rights of non‐English proficient people were being violated in local criminal courts simply because the courts didn't have language interpretation services. Initial meetings about what to do about this ranged widely, with a consensus to work with the local judges. But there was a problem with this strategy. Each criminal court judge in Orleans Parish (the county seat that is also contiguous with the City of New Orleans) is elected, and each bench on which the judge sits is her own domain. In other words, one judge on one bench cannot mandate another judge on another bench's administrative policies. This meant that to have system‐wide traction in Orleans Parish on the issue of language access within criminal courts, one would have to get all the local criminal court judges on board, as each judge's bench is its own closed system.

What cannot be assumed in any space, be that in a classroom, a corporate conference room, or a community meeting, is that people are aware of the disparate yet interlocking systems that influence the desired outcome they seek. One must be trained to build systems thinking habits in order to perceive these relationships, and then one must practice engaging with their complexities to build up the ability to consider, plan, and navigate within one's immediate reality with more clarity (Ericsson et al. 1993; see also Niewoehner‐Green et al., this issue). This was true of even this group of seasoned professionals, most of them practicing attorneys, who spent a considerable amount of our initial research and planning time contemplating how to get all thirteen Orleans Parish judges to cooperate on language access. During this phase of the work, no mapping or engagement work had been adequately conducted yet.

In this scenario, deeper analysis of the relevant systems (prompted by asking the simple question about what levers were in place that could be used to influence these thirteen elected judges at the same time), in which criminal court was either embedded or connected with, yielded a better approach. The group needed to adopt an inquiry mindset capable of asking more powerful questions that enabled them to move beyond the local systems in which they were deeply engaged and knowledgeable (Kaser and Halbert 2017). Frustration from the sheer seeming impossibility of wrangling 13 judges into agreement almost killed our momentum. The group needed to adapt by considering ideas beyond their normal habits and practices.

Instead of wasting additional energy attempting to win over 13 judges (equaling 13 separate systems), the suggestion was offered to target the Louisiana Supreme Court, which has the authority to mandate certain administrative practices for all judges across the State of Louisiana. This entity could yield the desired outcome (not to mention greater potential for system‐wide impact across the entire State of Louisiana), but it felt more out of reach than local judges. For the group to pivot and target the Louisiana Supreme Court, they needed to adapt to a new potential, one in which they perceived themselves as capable of affecting the highest court system in the state.

One of the attorneys who worked for a significant private law firm in New Orleans had deep relationships with the Louisiana State Bar Association. She engaged in leadership and leveraged this relationship into an ally on the issue. Another member of the group with ties to nationally certified court interpreters connected the group to conversations with field experts in different state systems who had initiated similar efforts. With new knowledge and allies in hand, this group of dedicated, but ultimately not very powerful people, created a plan of action that was followed through until arriving at a recommendation memo for a certified court interpretation standard for the Louisiana Supreme Court in 2010, which the agency reviewed positively, creating a committee in 2011 to evaluate action recommendations.

The proposed solution of an interpreter standard connected several systems (national court interpreter groups, the State of Louisiana Supreme Court and lower courts, criminal justice and language access advocates from hurricane‐ravaged regions, and the Louisiana State Bar Association) on one topic—due process for limited English proficient people in Louisiana courts. The solution was directly informed by community experiences in the courts at the local level and supported along the way by key partners both within and outside of the state. In 2025, an entire office at the Louisiana Supreme Court is dedicated to language access, now offering certified court interpreters in more than 15 languages (Louisiana Supreme Court n.d.), bringing to full realization the potential future outcome envisioned in 2009 when this group began its work.

### 2.3 Field Reflections

People's ability to examine a broader set of systems in both examples was largely aided by each group's community organizing capacities to engage, map, and adapt with interlocking systems. Consider the type of public impact both efforts achieved across systems. In the Road Home example, local requests for support would have pitted New Orleans against other parishes, likely having a negative effect on desired outcomes. In the court interpreter example, local work with elected officials may have yielded some judges implementing better language access processes and policies, but only within the bench of each judge in agreement. Both groups not only saw the interconnectivity across systems but also viewed how different networks created bridges across systems, which they tapped and mobilized toward their desired ends.

Although no one involved in the two cases described here would say that it was their practice in systems thinking approaches that played a key role in their efforts, I can retrospectively assert that this was indeed the case. Both cases researched and explored the complex relationships among different levels of systems and the leadership networks that interacted with these (Garcia and O'Driscoll 2020). They mapped, engaged actors in those systems, and adapted according to the information they learned. Advocates developed strategies informed by an expanded awareness of interlocking governance systems processes and touchpoints—abilities leadership educators should be keen to explore with their students (Day and Dannhäuser 2024).

## 3 Global Sustainable Development Field Notes

Akin to community‐based organizing at local levels, the world of international development requires more than a linear, program‐only approach, which often fails to address the intricate challenges of complex global landscapes effectively. Creating dynamic solutions involves a blend of curiosity, relatability, and a focus on optimal outcomes for people, while accounting for cultural complexities and change, which I (Dwan) demonstrate in my field notes from my experiences as a Peace Corps volunteer, and subsequently as a manager in the volunteer recruitment division.

In global sustainable development, systems thinking practices and approaches foster holistic strategies that acknowledge and respect local cultures and practices. As Senge (2006) noted, “In a world characterized by increasing interdependence, problems that were once distinct and separable become increasingly systemic” (p. 6). This systemic interconnectedness, which I witnessed firsthand, necessitates that leaders develop the capacity to see beyond simple cause‐and‐effect relationships and recognize the dynamic interplay of forces within a system. In my experiences, when such an approach is not applied in global sustainable development, the inadequacy of top‐down, project‐based interventions fails to account for the intricate web of relationships, feedback loops, and emergent properties that define community realities, often resulting in unsustainable projects.

### 3.1 English in Mongolia

My journey into global sustainable development was formed mainly as a Peace Corps Volunteer (PCV) in Mongolia from 2007 to 2009. While there, I taught English as a foreign language (TEFL) and supported a local water quality monitoring project handed down by the previous iteration of PCVs in partnership with The Asia Foundation. The Peace Corps employs a full‐immersion approach, allowing new Trainees to live with a host family and participate in a range of orientation events during their first 3 months of pre‐service training (PST—a formal Peace Corps process). Once trainees arrive in‐country, they spend a few days together prior to dividing into sectors and departing for their host communities, where they will live with host families while attending formal intercultural technical courses for 3 months. Formal checkpoints throughout this time include a site placement announcement event, which involves a trip to Ulaanbaatar, Mongolia's capital, to meet with counterparts with whom they'll work and live for the next 2 years. This is followed by a formal swearing‐in process hosted by the local Peace Corps office and host country dignitaries. The event features cultural performances and honors the rich cultural influences of the country.

As we explore cultures beyond textbooks and theories, and connect people through cultural practices, it becomes a living laboratory for systems‐thinking leadership through engagement. It was not just a job; it was deliberate, immersive training in adaptation and radical humility—a philosophy woven into every aspect, from PST to living with a host family for 3 months. This provides a foundational understanding of the engagement practice of cultural immersion into a foreign system's daily rhythms, its unspoken rules, and its deeply ingrained mental models and adaptation, which profoundly shaped my leadership approach. The constant, and sometimes uncomfortable, exposure with ways of being different than mine cultivated a divergent way of thinking, allowing me to see interdependence within a whole, a foundational cognitive shift that classrooms can aim to replicate with systems leadership concepts that explore how creativity plays a role in seeing systemic interactions (Puccio et al. 2010).

This profound immersion immediately highlighted critical leverage points within communities that external linear interventions often miss. My initial role as an education volunteer evolved into an eight‐and‐a‐half‐year commitment as a Peace Corps staff member from 2010 to 2019, including positions as a Regional Recruiter, Regional Recruitment Supervisor, and Staging Facilitator, which required me to work with staff in‐country around the world bridging volunteer training, expectations, and mental preparation to their respective country of service.

My goal as a Peace Corps Volunteer was to work myself out of a job within my two‐and‐a‐half‐year term, which I perceive as a direct application of adaptability, engagement, and expanded mental model approaches that enabled me to recognize that true sustainability lays in strengthening local capacity. I was a guest in these communities, after all, working alongside men and women who possessed far greater knowledge about their villages, their communities, and the needs of their children than I did.

By serving, I learned how to put engagement approaches to work, enabling me to experience the power of cultivating collectivism and building coalitions when solving problems for and with local communities while working with teachers across grades at the local school in my town of Khar‐Khorin, the old capital of Mongolia. Cultivating deep relationships and a nuanced understanding of my teachers’ experiences were critical components of this process. These teachers often had to identify where to test the system and where to push the envelope for mindset and culture change. In the area of teaching English, teachers wanted to create lasting generational change while remaining sensitive to potential issues of cultural dialect erosion. I collaborated with these teachers to balance teaching traditional Mongolian practices for young language learners with integrating a curriculum that preserved their heritage. It was a lesson in adaptability for all of us, as we co‐navigated these tensions in real time.

While there, I gained a deep appreciation for how and why the Peace Corps designs its training to account for the various phases of maturity development in a volunteer's experience. The first year is often about self‐discovery and adaptation, while subsequent in‐service training phases focus on empowering the Peace Corps Volunteer and their host‐country counterparts to build sustainable projects.

### 3.2 Cultural Systems in Haiti

Through my personal experiences as a volunteer, as well as serving as a staff person supporting other volunteers, I learned to value the need for a Peace Corps Volunteer to spend at least a year understanding their new context, becoming familiar with their community, experiencing life at the local level, and grasping the importance of studying and using the local language. However, it is the larger shift toward navigating differences and learning new systems where the Peace Corps truly engages in systems leadership development.

In Grand‐Bois, Haiti, where I worked for ServeHAITI (a Haitian‐based NGO) in the labor and delivery unit at a medical clinic, they were often challenged by the need to incorporate cultural nuances into clinical practices to accommodate the traditional practices of new mothers and their families. Welcoming new life while managing a steady volume of patients required the ability to perceive beyond the immediate clinical contexts. For example, this clinic added a space after delivery in the labor and delivery area, which led to an increase in the clinic's utilization, effectiveness, and trust in providing child and maternal care medical services.

In the clinic experience, everyone had to work together to address sustainable cultural practices, minimizing the footprint of outside cultural influences, and developing solutions with local doctors and patients. This practice is dynamic and iterative, requiring adaptability in real‐time, as the needs of surrounding communities and villages dictate the medical landscape. In this sense, the complex and diverse cultural systems that exist throughout the communities served by this clinic and the English language program in Mongolia required the project personnel to understand and subsequently incorporate this knowledge into their program practices.

This taught me that to engage a system effectively, you must first understand it from within its existing cultural boundaries, truly seeing and hearing everyone. Hence, the rich practice of community mapping (see Burns et al. 2012 for a sample mapping tool) enables an understanding of the systems, its actors, and their relationships as they influence systems dynamics, feedback loops, and transformative levers. In my experiences I have consistently sought to bring together diverse actors—including community members, government agencies, non‐profit organizations, and the private sector—to work collaboratively toward shared goals.

### 3.3 Field Reflections

For anyone involved in global systems thinking, the work and the experience inevitably lead to existential changes within the practitioner. However, these experiential growth phases in leadership are not always accounted for or discussed in traditional models. It's essential to bring forward the humanity of leaders in this work, emphasizing that success is deeply intertwined with a person's proximity to the people they serve, their cultural understanding of the challenges being addressed, and the nature of the relationships with colleagues from different cultural systems and experiences, emphasizing the value of an experiential approach to global development.

My experiences in the Peace Corps suggest a more holistic, systems‐oriented approach, emphasizing analytical skills, emotional intelligence, cultural competence, and the ability to adapt to change created through classroom learning, real‐world practice, and the application of cultural humility. This combination profoundly affected my ability to truly see and understand the systems in which I worked, enhancing my ability to identify the most effective levers for change. These experiences significantly shaped my perspective on problem‐solving and leadership. Although I didn't use the term “systems leadership” during my Peace Corps experiences and beyond, I recognize that living and working as a person capable of perceiving interlocking systems (Meadows 2008) at an early age profoundly changed how I navigated life and chose professional projects and roles.

## 4 From the Field to the Classroom: Considerations for Teaching

Many systems thinking models and practitioners emphasize the value of deep cultural understanding, relationship building, and experiential learning. For example, scholars like Edgar Schein (Schein and Schein 2017) and Peter Senge (2006), who work on organizational culture and learning organizations, highlight the importance of understanding the underlying assumptions and values that drive behavior within a system and the need for leaders to foster open communication and mutual respect. Similarly, the work of Humberto Maturana and Francisco Varela (1987) on the biology of cognition emphasizes the role of the observer in shaping their understanding of the system and the importance of recognizing the interconnectedness of all elements within a system.

In our combined field notes we identified several key concepts that a systems leadership framework promotes:

● Adaptiveness—being able to navigate complex and unpredictable situations, often with limited resources and unfamiliar cultural contexts (Walker et al. 2004).
  ○ Teaching pedagogy: combine case study with role play exercises that tackle complex issues
● Engagement—valuing a wide range of stakeholder views by actively engaging and prioritizing the building of strong, trusting relationships with people who occupy different parts of a system or operate across vastly different social systems.
  ○ Teaching pedagogy: assign engagement design projects for change work scenarios
● Mapping—Utilizing systems mapping techniques to visualize the complex interrelationships between different elements within a system. As Senge (2006) notes, “systems thinking is a discipline for seeing wholes. It is a framework for seeing interrelationships rather than things, for seeing patterns of change rather than static ‘snapshots’” (p. 68–69).
  ○ Teaching pedagogy: have students map players and power in real‐world interlocking systems on a given issue.
● Expanded mental models—the deeply ingrained assumptions and beliefs shaping a person's world perception requires questioning and reflection.
  ○ Teaching pedagogy: consider using guest speakers, field visits, and non‐traditional readings and reflection to create opportunities for mental model expansion

Integrating systems leadership components into practice has the potential to dramatically enhance a student's ability to collaborate effectively across cultures and to drive sustainable change within complex systems. The field notes offered here offer some insights into how systems leadership can come to life for students. Central to this approach is experiential learning, an influential strategy educators can use to create simulations, case studies, and offer community‐engaged opportunities (Dewey 1938; Kolb 1984). Educators can deepen students' understanding and foster critical thinking and problem‐solving skills by applying systems thinking approaches to real‐world scenarios—such as mapping stakeholder roles in local systems or designing social problem interventions that make use of systems leadership approaches.

Educators can model flexibility, openness to feedback, and a willingness to experiment with various teaching and learning approaches, which create a safe learning environment where students feel encouraged to take risks, learn from failures, and adjust their strategies in response to new information. Students can be empowered with systems leadership approaches that map practical systems, such as the community organizing power map or other community mapping tools used by communities. These tools are invaluable for helping individuals visualize complex systems, identify leverage points for change, and understand the potential consequences of interventions.

Finally, storytelling and narrative are powerful tools for teaching and learning. Educators can utilize personal and shared narratives to illustrate systems leadership concepts in practice, making them more relatable to students, particularly when real people enter the classroom and share the risks faced and how they met those risks. Sharing real‐world examples of systems thinking, whether successful or not, helps students grasp the complexities and nuances of this approach. Educators can instill this mindset by encouraging students to think beyond immediate solutions and consider the long‐term ramifications of their actions within the complex interactions of various systems.

The classroom can, indeed, offer various strategies that approximate the risks that come into play in the real world when systems are considered or ignored. Through a variety of pedagogical strategies that combine classroom instruction with experiential learning and reflection, students’ capacities to perceive and engage in systems leadership can be achieved.

## 5 Conclusion

In this article we argued how vital integrating systems leadership into leadership education is when preparing students who are capable of navigating increasingly complex and interconnected societal structures. We drew from our combined backgrounds in community organizing and sustainable development to show how a systems mindset in the field led to successes in complex situations. By incorporating real‐world complexity of interconnected systems via various experiential pedagogies, educators can better prepare students for effective collaboration and sustainable change.
